# Efficacy and safety of treating chronic nonspecific low back pain with radial extracorporeal shock wave therapy (rESWT), rESWT combined with celecoxib and eperisone (C + E) or C + E alone: a prospective, randomized trial

**DOI:** 10.1186/s13018-021-02848-x

**Published:** 2021-12-04

**Authors:** Xuejiao Guo, Lin Li, Zhe Yan, Yunze Li, Zhiyou Peng, Yixin Yang, Yanfeng Zhang, Christoph Schmitz, Zhiying Feng

**Affiliations:** 1 Department of Pain Medicine, The First Affiliated Hospital, Zhejiang University School of Medicine, Hangzhou, China; 2 Department of Anesthesiology, Yuyao People Hospital of Zhejiang, Ningbo, China; 3 Extracorporeal Shock Wave Research Unit, Chair of Neuroanatomy, Institute of Anatomy, Faculty of Medicine, LMU Munich, Munich, Germany

**Keywords:** Radial extracorporeal shock wave therapy (rESWT), Celecoxib, Eperisone, Chronic nonspecific low back pain

## Abstract

**Background:**

To investigate whether respectively radial extracoporeal shock wave therapy (rESWT) or a combination of rESWT, celecoxib and eperisone (rESWT + C + E) are superior in reducing pain in patients with chronic nonspecific low back pain (cnsLBP) compared to C + E alone (a standard treatment of this condition in China).

**Methods:**

140 patients with cnsLBP were randomly allocated to rESWT (*n* = 47), rESWT + C + E (*n* = 45) or C + E alone (*n* = 48) for four weeks between November 2017 and March 2019. Outcome was evaluated using the Pain Self-Efficacy Questionnaire (PSEQ), Numerical Rating Scale (NRS), Oswestry Low Back Pain Disability Questionnaire and Patient Health Questionnaire 9, collected at baseline as well as one week (W1), W2, W3, W4 and W12 after baseline.

**Results:**

All scores showed a statistically significant improvement over time. The PSEQ and NRS scores showed a significant Time × Treatment effect. Patients treated with rESWT had significantly lower mean NRS values than patients treated with rESWT + C + E at W1 and W3, as well as than patients treated with C + E alone at W3 and W4. No severe adverse events were observed.

**Conclusions:**

rESWT may not be inferior to respectively rESWT + C + E or C + E alone in reducing pain in patients with cnsLBP. Level of Evidence: Level I, prospective, randomized, active-controlled trial.

*Trial registration*: Clinicaltrials.gov Identifier NCT03337607. Registered November 09, 2017, https://www.clinicaltrials.gov/ct2/show/NCT03337607.

**Level of evidence:**

Level I; prospective, randomized, controlled trial.

**Supplementary Information:**

The online version contains supplementary material available at 10.1186/s13018-021-02848-x.

## Background

Nonspecific low back pain (nsLBP) is defined as low back pain not attributable to a recognizable, known specific pathology [1, 2]. The reported point prevalence of nsLBP is as high as 33% [2, 3], and its one-year prevalence is as high as 73% [2, 4]. Furthermore, its lifetime prevalence exceeds 70% in most industrialized countries [1], with an annual incidence of 15–20% in the USA [5]. In China, nsLBP has become one of the leading causes of disability-adjusted life-years in 2010 [6]. The prevalence of nsLBP was reported as 41% in Chinese adolescents [7].

Management of nsLBP is challenged by the problems that most back pain has no recognizable cause (> 85%), an underlying systemic disease is rare, and most episodes of back pain are unpreventable [2, 5, 8]. Risk factors for the development of disabling chronic nsLBP (cnsLBP) include preexisting psychological distress, disputed compensation issues, other types of chronic pain and job dissatisfaction [8–10].

In a recent consensus statement the Chinese Association for the Study of Pain (CASP) has considered nonsteroidal anti-inflammatory drugs (NSAIDs) (including the selective Cox-2 inhibitor celecoxib), skeletal muscle relaxants (SMRs) (including eperisone) and rehabilitation (including exercise therapy, physiotherapy and psychotherapy) as first-line treatments of cnsLBP [11].

Extracorporeal shock wave therapy (ESWT) is a safe and effective non-invasive treatment option for tendon and other pathologies of the musculoskeletal system [12–15]. During the last years a number of studies indicated that ESWT may also serve as an alternative in treatment of cnsLBP [16–26] (an overview on these studies is provided in Additional file 1). However, in none of these studies ESWT was compared with NSAIDs and SMRs, let alone the investigation of potential advantages of the combination of ESWT with NSAIDs and SMRs over respectively ESWT or NSAIDs and SMRs alone. In consequence, these studies are of limited help to further corroborate or challenge the aforementioned consensus statement by the CASP [11].

Considering the high incidence of cnsLBP, the limited evidence on which the aforementioned consensus statement by the CASP [11] was based and the limited relevance of all studies on treatment of cnsLBP with ESWT that were published so far [16–26], it was the aim of this study to test the following hypotheses in a prospective, randomized, active-controlled trial: (i) treatment of cnsLBP with radial ESWT (rESWT) is safe; and (ii) both rESWT and the combination of rESWT, celecoxib and eperisone (rESWT + C + E) are superior to celecoxib and eperisone (C + E) alone in treatment of cnsLBP.

## Methods

### Study design

**Fig. 1:**
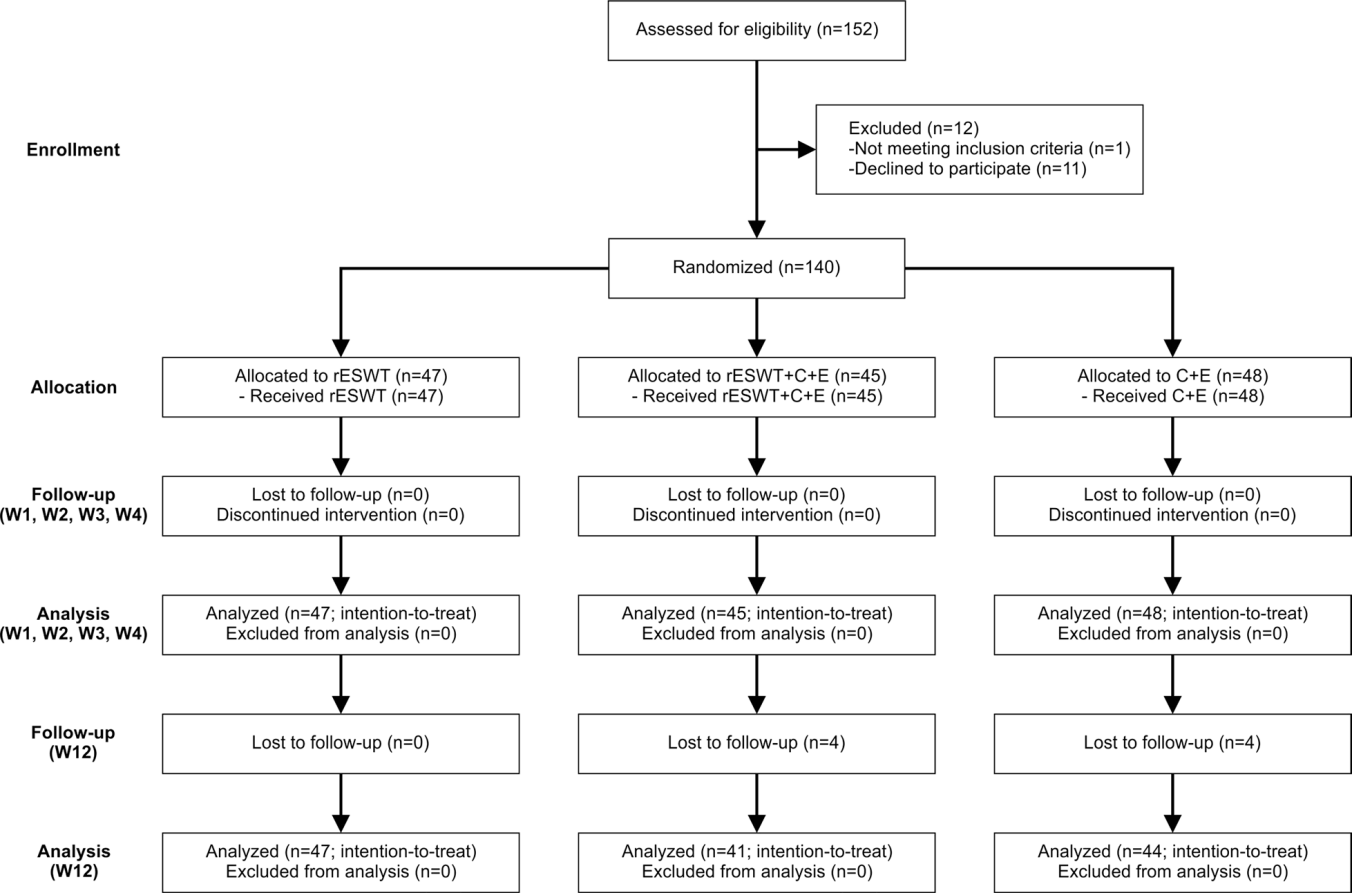
Flow of patients in this study according to the Consolidated Standards of Reporting Trials (CONSORT) statement [27]

This prospective, randomized, active-controlled trial was performed at the Department of Pain Medicine of the First Affiliated Hospital, Zhejiang University School of Medicine, Hangzhou, China (hereafter: "our department"). All patients were from the city of Hangzhou and from other cities in Zhejiang province (China). Figure 1 shows the flow of patients through this study according to the Consolidated Standards of Reporting Trials (CONSORT) statement [27], and Table 1 the schedule of enrollment, interventions and assessments according to the Standard Protocol Items: Recommendations for Interventional Trials (SPIRIT) [28].

**Table 1:** Schedule of enrollment, interventions and assessments during this study according to SPIRIT [28]

Study period					
	Enrollment/allocation	Post-allocation	Follow-up		Close-out
Timepoint	D0 (= BL)	D0 (= BL)	W1, W2, W3	W4	W12
Enrollment					
Clinical evaluation	X				
Eligibility screen	X				
Allocation	X				
Interventions					
rESWT		X	X		
C + E		X	X		
Assessments					
A, G, BH, BW, Du, ES, MS, Co	X				
Safety		X	X	X	X
PSEQ score	X		X	X	X
NRS score	X		X	X	X
OLBPDQ score	X		X	X	X
PHQ-9 score	X		X	X	X

D0, day zero; BL, baseline; W, week; rESWT, radial extracorporeal shock wave therapy; C + E, celecoxib and eperisone; A, age; G, gender; BH, body height; BW, body weight; Du, duration, ES, education status; MS, marriage status; Co, comorbidities; PSEQ, Pain Self-Efficacy Questionnaire; NRS, numerical rating scale; OLDPDQ, Oswestry Low Back Pain Disability Questionnaire; PHQ-9, Patient Health Questionnaire 9

### Ethics committee approval

This study was approved by the Ethics Committee of the First Affiliated Hospital, Zhejiang University School of Medicine, Hangzhou, China (no. 2017-515, dated 27/07/2017) and was carried out in accordance with the World Medical Association Declaration of Helsinki. Patients were allowed to withdraw the free and informed consent term to participate in the study at any time. The study was registered with ClinicalTrials.gov (Identifier NCT03337607) [29].

### Participants

A total of *n* = 152 patients suffering from cnsLBP were assessed for eligibility to be enrolled in this study between November 2017 and March 2019. Diagnosis was based on the patient's history, physical examination at our department and X-rays of the lumbar spine. Patients were considered for participation in this study according to the inclusion and exclusion criteria summarized in Table 2.

**Table 2:** Inclusion and exclusion criteria of patients with chronic nonspecific low back pain enrolled in this study

Inclusion criteria
Adults (both male and female) with nonspecific low back pain for more than three months
Age range: between 18 and 80 years
Willingness of the patient to participate in the study, and written informed consent signed and personally dated by the patient
Chronic nonspecific low back pain clinically diagnosed as repeated lumbar sourness and swelling pain or a chronic progressive process, accompanied by (i) X-ray examination to exclude lumbar vertebrate fractures, spondylolysis, spondylolisthesis and severe osteoporosis, and/or (ii) MRI with normal signal or low nucleus pulposus signal
No contraindications of rESWT or treatment with celecoxib and eperisone
Exclusion criteria
Children and teenagers below the age of 18
Elderly aged > 80 years old
No willingness of the patient to participate in the study, and/or written informed consent not signed and not personally dated by the patient
Previous spinal fracture or spinal surgery
Protrusion of a lumbar intervertebral disk, ankylosing spondylitis, scoliosis, lumbar spondylolisthesis and lumbar spondylolysis
Systemic disorders and psychiatric disorders
Contraindications of rESWT (pregnancy, presence of blood-clotting disorders (including local thrombosis), presence of local tumors, local bacterial and/or viral infections (including lumbar vertebral tuberculosis), treatment with oral anticoagulants and/or local corticosteroid applications in the period of six weeks before the first rESWT session (if applicable)
Contraindications of treatment with celecoxib and eperisone (allergy to celecoxib, eperisone or sulfonamides, presence of gastrointestinal bleeding or bleeding history, renal dysfunction and/or severe heart failure, and lactating women)
Participation in any other clinical trial in the period of 12 weeks before potential inclusion in this study

Before randomization, a thorough explanation of the various options, as well as the potential risks, benefits and outcomes associated with the various options, took place. A total of 12 patients assessed for eligibility were excluded because they did not meet the inclusion criteria (one patient) or the patients chose to withdraw or declined to sign the consent form (eleven patients). After having obtained written informed consent from each of the remaining *n* = 140 patients, they were randomly assigned to receive respectively rESWT (*n* = 47), rESWT + C + E (*n* = 45) or C + E alone (*n* = 48).

### Randomization and blinding

The randomization plan of this study was generated by Y.L. and Z.P. before the first patient was enrolled in this study. Specifically, in line with the estimated enrollment of 150 patients specified in the protocol of this study [29] a randomization plan was generated using a computerized random number generator [30] (three treatment labels (rESWT, rESWT + C + E, C + E alone), 150 subjects, five blocks). The results of this procedure were kept in 150 consecutively numbered, sealed, opaque envelopes, with a note of the corresponding treatment label in each envelope. For each patient who was screened and who signed the informed consent form, the next envelope was used to allocate the patient to either of the treatment groups. Accordingly, allocation was kept concealed to both the patient and the therapists until the patient's treatment started.

**Fig. 2:**
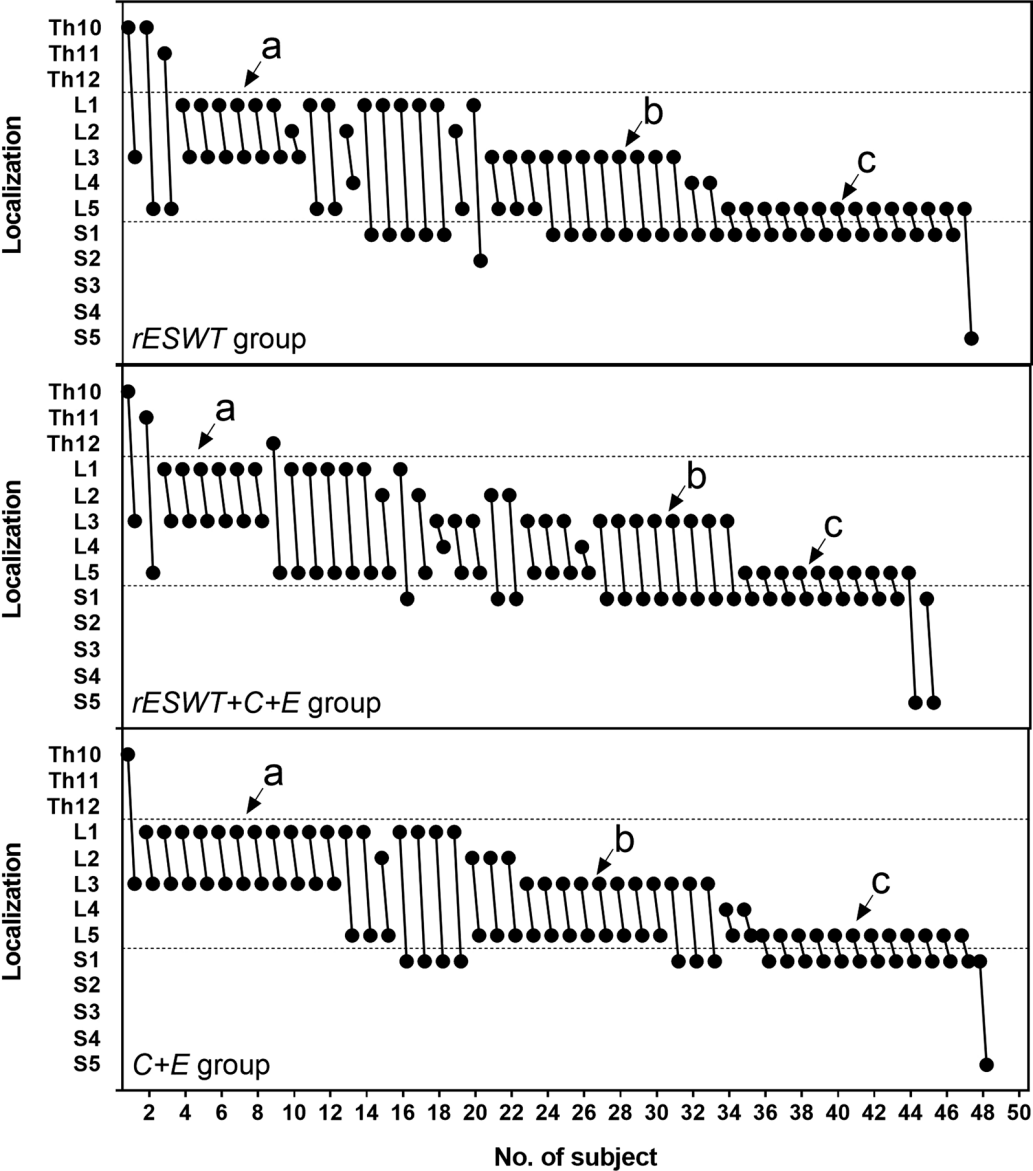
Individual localization of chronic nonspecific low back pain reported by the patients enrolled in this study. The arrows exemplarily indicate patients who reported pain from the first lumbar segment of the spine (L1) to the third lumbar segment of the spine (L3) (**a**), from L3 to the first sacral segment of the spine (S1) (**b**) or from the fifth lumbar segment of the spine (L5) to S1 (**c**), respectively

Characteristics of included patients at baseline are summarized in Table 3; individual localization of cnsLBP is displayed in Fig. 2.

**Table 3:** Characteristics of included patients at baseline (intention-to-treat population)

Variable\group	rESWT (*n* = 47)	rESWT + C + E (*n* = 45)	C + E (*n* = 48)
Age, years, median; mean (SD; min; max)	34; 34.9 (8.7; 21; 58)	34; 36.5 (10.8; 21; 64)	32; 36.0 (11.2; 21; 70)
Woman, n (%)	22 (46.8)	28 (62.2)	25 (52.1)
Body weight, kg, median; mean (SD; min; max)	60; 62.3 (11.2; 41; 82)	58; 60.8 (11.3; 40; 82)	67; 64.4 (10.2; 42; 80)
Body height, cm, median; mean (SD; min; max)	167; 166 (7.3; 153; 185)	161; 163 (7.6; 148, 180)	163; 165 (8.9; 130; 183)
BMI, kg/m^2^, median; mean (SD; min; max)	22.6; 22.3 (3.0; 16.7; 28.3)	22.3; 22.7 (3.2; 17.0; 31.2)	23.8; 23.7 (3.7; 17.0; 41.4)
Duration^a^, months, median; mean (SD; min; max)	12; 33.5 (45.8; 3, 240)	24; 29.6 (30.2; 3; 120)	12; 29.9 (49.1; 3; 240)
Education status^b^	0; 1; 7; 39	0; 8; 7; 30	1; 5; 15; 27
Marriage status^c^	7, 39, 1, 0	9, 36, 0, 0	7, 40, 1, 0
Comorbidities, n (diagnosis)	1 (diabetes)	0	1*
PSEQ score, median; mean (SD; min; max)	55; 51.3 (8.5; 26; 60)	50; 46.8 (12.0; 8, 60)	53.5; 48.8 (10.9; 17; 60)
NRS score, median; mean (SD; min; max)	4; 4.2 (1.2; 1; 6)	4; 4.4 (1.4; 1; 7)	4; 4.2 (1.5; 2; 8)
OLDPDQ score, median; mean (SD; min; max)	14.0; 16.8 (11.4; 2; 66)	20.0; 21.6 (12.8; 4; 68)	18.0; 18.9 (10.4; 0; 50)
PHQ-9 score, median, mean (SD; min; max)	3; 4.6 (4.1; 0; 14)	5; 6.2 (5.1; 0; 22)	6; 6.9 (4.7; 0; 19)

^a^Duration of symptoms before enrollment into this study

^b^0, no education; 1, pupil; 2, middle school; 3, higher education

^c^1, not married; 2, married; 3, divorced; 4, widow. *, Sjogren's syndrome

SD, standard deviation; min, minimum value; max, maximum value; BMI, body mass index; PSEQ, Pain Self-Efficacy Questionnaire; NRS, numerical rating scale; OLDPDQ, Oswestry Low Back Pain Disability Questionnaire; PHQ-9, Patient Health Questionnaire 9

The protocol of this study [29] did not allow to blind the patients and the therapist who performed the treatments (L.L.). On the other hand, the person who performed all baseline and follow-up evaluations (X.G.) as well as all other researchers involved in this study (Z.Y., Y.Y., Y.Z., C.S and Z.F.) were blinded for the entire duration of the study. Specifically, X.G. and all other researchers involved in this study did not have access to the patients’ treatment records, including patient allocation or the allocation sequence, until all patients had completed the last follow-up examination.

### Interventions

Patients in the rESWT group received rESWT using the Swiss DolorClast device (Electro Medical Systems, Nyon, Switzerland) and the EvoBlue handpiece. Each patient received four rESWT sessions (one session per week). Each session consisted of a total of 4000 radial extracorporeal shock waves (rESWs) (1000 rESWs each applied to the left and the right paravertebrate muscles from the third lumbar segment (L3) to the first sacral segment (S1) using the 36-mm applicator, plus 1000 rESWs each applied to the left and the right sacroiliacal joint using the 15-mm convex applicator, in prone position of the patient). The air pressure of the rESWT device (and, thus, the energy flux density (EFD) of the applied rESWs) was gradually increased during the first 200 rESWs each until the maximum discomfort the patient could tolerate was reached, followed by 800 rESWs at this air pressure/EFD. The rESWs were applied at a frequency of 15 rESWs/second. No anesthesia or analgesic drugs were applied during the rESWT sessions.

Patients in the rESWT + C + E group received rESWT as the patients in the rESWT group plus celecoxib (1 × 200 mg (mg) per day for moderate pain (Numerical Rating Scale (NRS) score 4–6) or 2 × 200 mg per day (NRS score 7–10)) and eperisone (3 × 50 mg per day) for four weeks.

Patients in the C + E group received celecoxib and eperisone as the patients in the rESWT + C + E group, but no rESWT.

In addition, all patients were advised to perform simplified, safe core stability training and flexion/relaxation training at home, which was mainly based on the contraction of the lumbar muscles, under the guidance of a unified rehabilitation training video (two training sessions per week; each training session lasting for approximately 20 min; training for the entire duration of the study).

### Outcome measures and assessments

The primary outcome measure was change in the Pain Self-Efficacy Questionnaire (PSEQ) score [31]. Specifically, patients were asked to rate how confident they were at the time of examination despite the presence of their pain in performing the activities described in Table 4, listed by selecting a number on a 7 point scale where 0 equals "not at all confident" and 6 equals "completely confident". Scores on the PSEQ may range from 0 to 60, with higher scores indicating stronger selfefficacy beliefs. A point score change of 11 points for the PSEQ score corresponds to the minimal clinically important difference (MCID), which is described in the literature as the smallest difference that patients and clinicians perceive to be worthwhile when treating cnsLBP [32]. According to the latter study the PSEQ score is responsive to clinically important change over time. Furthermore, the same study [32] indicated that based on receiver operating characteristic curve analysis, the PSEQ was more responsive than the Numerical Rating Scale (NRS) and the Oswestry Low Back Pain Disability Questionnaire (OLDPDQ) to detect clinically important change in patients with chronic low back pain. The PSEQ score was collected at baseline (BL) and at all follow-up examinations, i.e., one week (W1), two weeks (W2), three weeks (W3), four weeks (W4) and twelve weeks (W12) after baseline.

**Table 4:** Activities assessed by the Pain Self-Efficacy Questionnaire (PSEQ) [31]

No.	Activity
1	I can enjoy things, despite the pain
2	I can do most of the household chores (e.g. tidying-up, washing dishes, etc.), despite the pain
3	I can socialize with my friends or family members as often as I used to do, despite the pain
4	I can cope with my pain in most situations
5	I can do some form of work, despite the pain (“work” includes housework, paid and unpaid work)
6	I can still do many of the things I enjoy doing, such as hobbies or leisure activitiy, despite the pain
7	I can cope with my pain without additional medication (next to study treatment)
8	I can still accomplish most of my goals in life, despite the pain
9	I can live a normal lifestyle, despite the pain
10	I can gradually become more active, despite the pain

In the protocol of this study treatment success was defined as individual improvement of the PSEQ score by more than 20 points at W12 (which is the median of the follow-up intervals used in the studies on ESWT for nonspecific low back pain summarized in Additional file 1).

Secondary clinical outcome measures were changes in the NRS score (patients were asked to rate their pain intensity on an 11-point scale where 0 indicated no pain at all and 10 indicated worst imaginable pain), the OLDPDQ score [33, 34] and the Patient Health Questionnaire 9 (PHQ-9) score [35]. The NRS, OLDPDQ and PHQ-9 scores were collected at BL, W1, W2, W3, W4 and W12.

Complications, adverse events and complaints during treatment were documented.

### Drop-outs and loss to follow-up

All patients received treatment as allocated. None of the 47 patients in the rESWT group, four out of the 45 patients in the rESWT + C + E group and four out of the 48 patients in the C + E group were lost to final follow-up at W12, resulting in full analysis of respectively 47/47 (100%) of the patients in the rESWT group, 41/45 (91.1%) of the patients in the rESWT + C + E group and 44/48 (91.7%) of the patients in the C + E group who were randomized.

### Power analysis

In all studies on ESWT for nsLBP that were published at the time of registering this study with ClinicalTrials.gov [20–23] no definition of treatment success was provided, no Power analysis was reported and the PSEQ score was not used to assess clinical outcome (c.f. Additional file 1). Therefore, these studies were only of very limited use for the purpose of performing a Power analysis of this study.

Based on anecdotal evidence from several therapists in Europe and Latin America who had used rESWT for treating cnsLBP for more than a decade we hypothesized that treatment of cnsLBP with rESWT as described above will result in a success rate of approximately 70%, and the combination of rESWT + C + E in a success rate of approximately 85%. Furthermore, based on our own experience we hypothesized that treatment of cnsLBP with C + E as described above will result in a success rate of only approximately 40%.

Considering a two-sided significance level of 95%, power of 0.8 and equal samples, the power analysis retrieved a minimum number of respectively *n* = 44 (according to [36]) or *n* = 42 (according to [37]) patients per group to be enrolled in this study (i.e., a minimum total number of *n* = 132 patients according to [36] or *n* = 126 patients according to [37]). The power analysis was performed with the online tool, Open Source Epidemiologic Statistics for Public Health [38].

Because of the low drop-out rate enrollment of patients was stopped after a total of *n* = 140 patients were enrolled in this study (i.e., before the estimated enrollment of 150 patients specified in the protocol of this study [29] was reached).

### Statistical analysis

Statistical analysis was performed on an intention-to-treat basis using the Last Observation Carried Forward Approach [39].

Mean and standard deviation (SD) were calculated for all investigated variables. The D’Agostino and Pearson omnibus normality test was used to determine whether the distribution of the investigated variables of the patients in the different treatment groups were consistent with a Gaussian distribution. According to [40] no significance tests of baseline differences were performed.

Treatment-related differences in mean PSEQ scores, mean NRS scores, mean OLDPDQ scores and mean PHQ-9 scores between the groups were tested with two-way repeated measures ANOVA, with the different follow-up times (BL, W1, W2, W3, W4 and W12) as within-patient factor and the different groups as between-patient factor. Comparisons between groups were performed using Bonferroni's multiple comparisons test.

In all analyses an effect was considered statistically significant if its associated P value was smaller than 0.05. Calculations were performed using GraphPad Prism (Version 9.0.0; GraphPad software, San Diego, CA, USA).

### Role of the funding source

This study did not receive any external funding. The funders of the study (i.e., the affiliations of the authors) had no role in , data collection, data analysis, data interpretation and writing the report. The corresponding author had full access to all the data in the study and had final responsibility for the decision to submit for publication.

## Results

No apparent differences between the groups were observed at baseline except for the mean PHQ-9 score (Table 3).

**Fig. 3:**
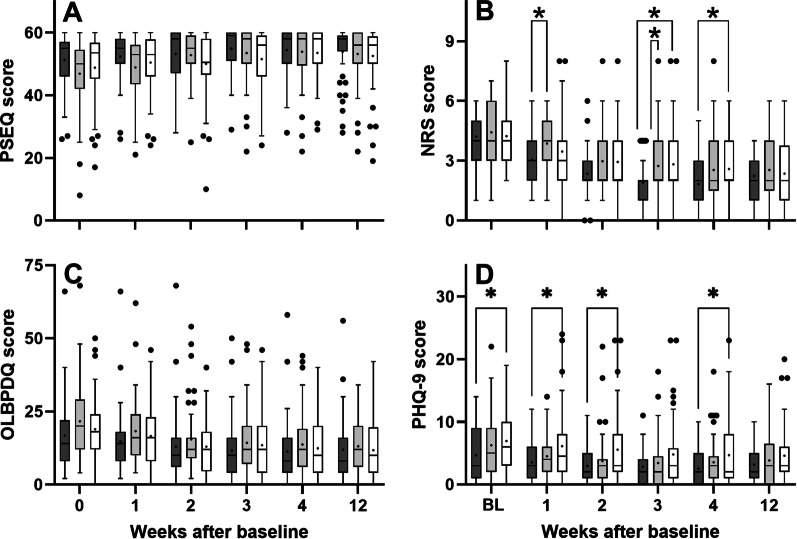
Tukey boxplots of (**A**) PSEQ score, (**B**) NRS score, (**C**) OLBPDQ score and (**D**) PHQ-9 score of patients suffering from chronic nonspecific low back pain who were treated with respectively rESWT (dark gray bars), rESWT + C + E (light gray bars) or C + E alone (open bars) at baseline (*X* = 0) and different follow-up times after baseline. Results of statistical analysis using Bonferroni's multiple comparison test are indicated (*, *p* < 0.05)

All investigated variables showed substantial interindividual variation and a statistically significant improvement over time (Fig. 3 and Table 5).

**Table 5:** Results (p values) of the statistical analysis of the data shown in Fig. 3 using two-way repeated measures ANOVA and Bonferroni's multiple comparisons test. P values < 0.05 are given boldface

ANOVA: source of variation	PSEQ score	NRS score	OLBPDQ score	PHQ-9 score
Time	** < 0.001**	** < 0.001**	** < 0.001**	** < 0.001**
Treatment	0.339	0.067	0.367	**0.027**
Time × Treatment	**0.044**	**0.016**	0.538	0.423
Patient	** < 0.001**	** < 0.001**	** < 0.001**	** < 0.001**

Bonferroni's multiple comparisons test	PSEQ score	NRS score	OLBPDQ score	PHQ-9 score
W0				
rESWT vs rESWT + C + E	0.063	> 0.999	0.091	0.217
rESWT vs C + E	0.561	> 0.999	> 0.999	**0.027**
rESWT + C + E vs C + E	0.920	> 0.999	0.646	> 0.999
W1				
rESWT vs rESWT + C + E	0.199	**0.027**	0.370	0.850
rESWT vs C + E	0.921	0.540	> 0.999	**0.011**
rESWT + C + E vs C + E	> 0.999	0.579	> 0.999	0.219
W2				
rESWT vs rESWT + C + E	> 0.999	0.130	0.830	> 0.999
rESWT vs C + E	0.331	0.163	> 0.999	**0.010**
rESWT + C + E vs C + E	0.443	> 0.999	0.786	0.126
W3				
rESWT vs rESWT + C + E	> 0.999	**0.029**	0.669	> 0.999
rESWT vs C + E	0.232	**0.010**	> 0.999	0.082
rESWT + C + E vs C + E	0.921	> 0.999	> 0.999	0.370
W4				
rESWT vs rESWT + C + E	> 0.999	0.077	0.863	0.755
rESWT vs C + E	> 0.999	**0.046**	> 0.999	**0.043**
rESWT + C + E vs C + E	> 0.999	> 0.999	> 0.999	0.608
W12				
rESWT vs rESWT + C + E	> 0.999	> 0.999	> 0.999	> 0.999
rESWT vs C + E	> 0.999	> 0.999	> 0.999	0.324
rESWT + C + E vs C + E	> 0.999	> 0.999	> 0.999	> 0.999

The PSEQ score showed a statistically significant Time × Treatment effect, with no significant difference between groups at any investigated time (Table 5). The number of patients with treatment success as defined in the protocol of this study at W1/W2/W3/W4/W12 after baseline was 1/0/1/2/2 among the patients in the rESWT group, 0/3/4/4/4 among the patients in the rESWT + C + E group and 2/3/2/4/4 among the patients in the C + E group. On the other hand, the number of patients who could not reach treatment success as defined in the protocol of this study because they had a PSEQ score > 40 at baseline was 41 (87.2%) among the patients in the rESWT group, 35 (77.8%) among the patients in the rESWT + C + E group and 39 (81.3%) among the patients in the C + E group. Furthermore, the number of patients who could not reach the MCID of 11 points because they had a PSEQ score > 49 at baseline was 31 (66.0%) among the patients in the rESWT group, 24 (53.3%) among the patients in the rESWT + C + E group and 29 (60.4%) among the patients in the C + E group.

The NRS score also showed a statistically significant Time × Treatment effect (Fig. 3 and Tables 5 and 6). Bonferroni's multiple comparisons test showed no statistically significant difference between the groups at baseline (rESWT: 4.2 ± 1.2; rESWT + C + E: 4.4 ± 1.4; C + E: 4.2 ± 1.5; all values given as mean ± SD) but between the patients in the rESWT group and those in the rESWT + C + E group at W1 after baseline (3.0 ± 1.2 vs. 3.9 ± 1.4) and W3 after baseline (1.9 ± 1.3 vs. 2.7 ± 1.4), as well as between the patients in the rESWT group and those in the C + E group at W3 after baseline (1.9 ± 1.3 vs. 2.8 ± 1.9) and W4 after baseline (1.8 ± 1.4 vs. 2.6 ± 1.7).

**Table 6:** Mean ± standard deviation of the NRS score before and at different times after baseline in this study

Treatment	rESWT		rESWT + C + E		C + E	
Baseline	4.2 ± 1.2		4.4 ± 1.4		4.2 ± 1.5	
W1/Ch	3.0 ± 1.2	− 27.8%	3.9 ± 1.4	− 12.6%	3.5 ± 1.9	− 18.2%
W2/Ch	2.3 ± 1.2	− 44.5%	3.0 + 1.4	− 32.7%	2.9 ± 1.8	− 30.5%
W3/Ch	1.9 ± 1.3	− 54.5%	2.7 ± 1.4	− 38.2%	2.8 ± 1.9	− 33.2%
W4/Ch	1.8 ± 1.4	− 56.6%	2.5 ± 1.6	− 42.7%	2.6 ± 1.7	− 38.9%
W12/Ch	2.2 ± 1.3	− 47.7%	2.5 ± 1.6	− 42.7%	2.4 ± 1.6	− 44.3%

W1/W2/W3/W4/W12, one/two/three/four/twelve weeks after baseline; Ch, change compared to baseline

The patients in the rESWT group had statistically significantly lower mean PHQ-9 scores than the patients in the C + E group at baseline (4.6 ± 4.1 vs. 6.9 ± 4.7), W1 after baseline (4.6 ± 4.4 vs. 6.9 ± 4.7), W2 after baseline (3.6 ± 3.0 vs. 6.1 ± 5.5) and W4 after baseline (2.9 ± 2.9 vs. 4.8 ± 5.4).

No severe adverse events were observed during this study.

## Discussion

This is the first study that compared the efficacy and safety of rESWT, rESWT + C + E and C + E alone in treatment of cnsLBP. Several conclusions can be drawn from the results of this study, some of which appear of relevance mostly to China, whereas others are of relevance to therapists worldwide.

### Assessment of outcome after treating cnsLBW with rESWT, rESWT + C + E or C + E alone using the Pain Self-Efficacy Questionnaire

A key finding of this study was that the PSEQ score [31] turned out to be unsuitable for evaluating treatment success in this study. This was due to the fact that 115 out of the 140 patients (82.1%) investigated in this study had a PSEQ score of 41 or higher at baseline and, thus, could not reach treatment success as defined in the protocol of this study. Furthermore, 83 out of the 140 patients (59.3%) had a PSEQ score of 50 or higher at baseline and, thus, could not reach a point score change of 11 points which has been defined in the literature as MCID of the PSEQ score when treating cnsLBP [32]. In this regard it is crucial to bear in mind that the patients investigated in this study were not specifically selected. Rather, they are representative for all patients who seek medical treatment of cnsLBP at our department. It is quite possible that in other countries, a substantial amount of these patients would have consulted their family doctor before visiting a department of pain medicine at a hospital affiliated with a university school of medicine. However, a family doctor system does not exist in China.

Until now the PSEQ score has not been used in any study listed in PubMed on treatment of cnsLBP performed at a department of pain medicine in China. One study on this topic authored by Chinese authors has been listed in PubMed so far [41]. The patients investigated in this study were recruited from the Rehabilitation Clinic of The Hong Kong Polytechnic University and were treated with respectively physiotherapy (Group 1) or physiotherapy + smartphone-based remote self-management (Group 2). The mean PSEQ scores at baseline in [41] (Group 1: 34.3 ± 8.0; Group 2: 38.6 ± 8.5; mean ± SD) were lower than the mean PSEQ score of the patients investigated in this study (49.0 ± 10.6). However, there are a number of substantial shortcomings in [41] which renders this study insufficient for contributing to evaluating the mean PSEQ score of patients with cnsLBP who seek treatment at a department of pain medicine in China: (i) the number of patients investigated in [41] was very small (Group 1: *n* = 3; Group 2: *n* = 5) and, thus, the total number of patients investigated in [41] (*n* = 8) was less than 20% of the number of patients in each group investigated in this study; and (ii) the only inclusion criteria reported in [41] were nonspecific low back pain due to musculoskeletal origins, access to a mobile phone and the ability to perform a brief exercise during regular working hours; and the only exclusion criterion reported in [41] was history of receiving major surgery. This is quite different to the inclusion and exclusion criteria used in this study (Table 2).

### Assessment of outcome after treating cnsLBW with rESWT, rESWT + C + E or C + E alone using the Numerical Rating Scale

Our finding of efficacy of rESWT for cnsLBP assessed using the NRS score (Fig. 3B) is in line with several reports in the literature on both rESWT [16, 17, 22, 23] and focused ESWT (fESWT) [18, 25, 26] (c.f. Additional file 1). On the other hand, there was no advantage in combining rESWT with C + E over respectively rESWT or C + E alone in treatment of cnsLBP in this study (to our knowledge studies on C + E alone in treatment of cnsLBP have not been published so far). Rather, when assessed using the NRS score rESWT was superior to rESWT + C + E in pain relief at W1 and W3 after baseline, and superior to C + E alone at W3 and W4 after baseline (Fig. 3B). No other study on rESWT or fESWT for cnsLBP published so far (c.f. Additional file 1) has addressed this important question about potential synergistic effects of combining rESWT or fESWT with pharmacological treatments in the management of cnsLBP.

With over 48.000 randomized controlled trials (RCTs), systematic reviews and clinical practice guidelines the PEDro database [42] is currently the largest independent database in the field of physical and rehabilitation medicine. All RCTs listed in the PEDro database are independently assessed for quality; all but two of the PEDro scale items are based on the Delphi list [43]. Currently, there are 1877 studies on low back pain listed in the PEDro database. Of note, only three out of these 1877 studies (0.16%) compared the efficacy of non-pharmacological, non-surgical treatments with pharmacological treatments and the combination thereof in the management of cnsLBP [44–46] (summarized in Additional file 2). One study on acute low back pain [44] compared spinal manipulation (two to three treatment sessions per week for up to four weeks) with paracetamol (4 × 1 g/day for up to four weeks) and the combination thereof and found no statistically significant reduction in the number of days to recovery of any of these treatments compared with placebo spinal manipulation and placebo drug treatment. Another study that comprised patients suffering from respectively acute low back pain, migraine or ankle sprain [45] compared a single session of acupuncture with pharmacotherapy (different protocols; summarized in Additional file 2) and the combination thereof and found that all of these strategies reduced mean pain by approximately 24% one hour after baseline (individual outcome for the different indications and longer follow-up times were not reported in [45]).

The third of these studies listed in the PEDro database [46] compared acupuncture (two treatment sessions per week for five weeks) with baclofen (a centrally acting skeletal muscle relaxant; [47]) (2 × 15 mg/day for five weeks) and the combination thereof in treatment of cnsLBP. The authors of this study found that both acupuncture and the combination of acupuncture and baclofen statistically significantly reduced the mean NRS score at both W5 and W10 after baseline (acupuncture: reduction by 30.2% at W5 after baseline and 21.9% at W10 after baseline; acupuncture + baclofen: reduction by 38.5% at W5 after baseline and 27.7% at W10 after baseline) but not baclofen alone. Furthermore, the combination of acupuncture and baclofen was statistically significantly more effective than acupuncture alone at W10 after baseline [46]. The percentage improvement in mean NRS score after treatment with acupuncture and baclofen at W5/W10 after baseline in [46] was 32%/42% lower than after treatment with rESWT at W4/W12 after baseline in this study. Based on these data one might speculate that rESWT could be superior to acupuncture in treating cnsLBP. This should be addressed in future studies (acupuncture was not used as control treatment in any of the studies on rESWT and fESWT in treatment of cnsLBP listed in Additional file 1).

### Assessment of outcome after treating cnsLBW with rESWT, rESWT + C + E or C + E alone using the Oswestry Low Back Pain Disability Questionnaire

The OLBPDQ score showed a statistically significant improvement over time (p < 0.001) but no statistically significant Time × Treatment effect (Table 5). In this regard it is of note that different MCID scores reported in the literature lead to different clinical prediction rules for the Oswestry disability index for the same sample of patients [48]. Table 7 summarizes the absolute and relative numbers of patients in the different groups in this study who reached the different MCID scores of the OLBPDQ score as described in [48] (comparing data obtained at baseline with data obtained at 12 weeks post treatment).

**Table 7:** Absolute and relative numbers of patients in the different groups in this study who reached the different MCID scores of the OLBPDQ score as described in [48] (comparing data obtained at baseline with data obtained at 12 weeks post treatment)

Group	1	2	3	1	2	3
	*n*_A_	*n*_A_	*n*_A_	*n*_R_	*n*_R_	*n*_R_
≥ 50% change from the initial to final OLBPDQ score	16	19	19	34.0	42.2	39.6
≥ 30% change from the initial to final OLBPDQ score	21	25	28	44.7	55.6	58.3
≥ 17-point decrease from the initial to final OLBPDQ score	2	9	6	4.3	20.0	12.5
≥ 10-point decrease from the initial to final OLBPDQ score	11	17	14	23.4	37.8	29.2
≥ 6-point decrease from the initial to final OLBPDQ score	19	27	27	40.4	60.0	56.3
≤ 20% final OLBPDQ score	39	39	44	83.0	86.7	91.7

Group 1, treatment with rESWT; Group 2, treatment with rESWT + C + E; Group 3, treatment with C + E alone; *n*_A_, absolute number, *n*_R_, relative number [%]

The smallest relative number of patients who reached the different MCID scores was obtained after treating cnsLBP with rESWT, which was most probably due to the lowest mean OLBPDQ score of the rESWT group at baseline (Fig. 3C). On the other hand, except for the MCID score " ≤ 20% final OLBPDQ score" the highest relative number of patients who reached the different MCID scores was obtained after treating cnsLBP with rESWT + C + E, which was most probably due to the highest mean OLBPDQ score of the rESWT + C + E group at baseline (Fig. 3C). In any case, these data did not justify to conclude any difference in the outcome of treating cnsLBP with rESWT, rESWT + C + E or C + E alone using the OLBPDQ in this study.

### Assessment of outcome after treating cnsLBW with rESWT, rESWT + C + E or C + E alone using the Patient Health Questionnaire 9

As in case of the OLBPDQ, the PHQ9 score showed a statistically significant improvement over time (p < 0.001) but no statistically significant Time × Treatment effect (Table 5). On the other hand, two-way repeated measures ANOVA demonstrated a statistically significant difference between the groups regarding the mean PHQ9 scores (*p*_Treatment_ = 0.027) (Table 5). As shown in Fig. 3 this was mostly due to statistically significant differences in the mean PHQ9 score between the rESWT group and the C + E group at baseline as well as one week, two weeks and four weeks after baseline. In this regard it remains unknown why despite randomization, the patients in the C + E group had a statistically significantly higher mean PHQ-9 score at baseline than the patients in the rESWT group. However, according to [40] no adjustment of this patients imbalance was performed (by, e.g., some form of multiple regression analysis).

### Molecular and cellular mechanisms of action of rESWT in treatment of chronic, non-specific low back pain

The question why in this study treatment of cnsLBP with rESWT was superior to treatment with rESWT + C + E or C + E alone (when assessed using the NRS), whereas in [46] treatment with acupuncture + baclofen was superior to treatment with either acupuncture or balcofen alone, cannot be fully answered at this time. Although no study has systematically addressed similarities and differences in molecular and cellular mechanisms of action between extracorporeal shock waves (ESWs) and acupuncture, it is reasonable to hypothesize that these mechanisms are not the same. To make matters worse, the mode of action of eperisone (used in this study) is different from the mode of action of baclofen (used in [46]). In cnsLBP ESWT most probably acts mainly via reduction of substance P and calcitonin gene-related peptide in C nerve fibers [49, 50]. Substance P nerve fibers were demonstrated within subchondral bone of degenerative lumbar facet joints in humans and hypothesized being involved in the etiology of low back pain [51]. Both substance P and CGRP were found in human cervical facet joint capsules [52]; substance P was also found in the lumbar facet joint capsule of rabbits [53] (c.f. also [54]). The high amount of substance P in active trigger points [55] as well as the ability of ESWs to impair acetylcholine receptors at the neuromuscular junction [56] and to increase the expression of lubricin in tendons and septa [57] (which improves the gliding ability of tendons and septa [58]) are additional factors that may contribute to the beneficial effects of ESWT in the management of cnsLBP.

It is well known that effects of ESWs on nociceptors can be blocked by local anesthesia [59] and, thus, treatment of tendinopathies with ESWT without local anesthesia is more effective than with local anesthesia [60, 61]. Eperisone is a centrally acting skeletal muscle relaxant which directly acts on motor nerves to hyperpolarize the action potential, resulting in reduction of nerve sensitivity and conduction of the muscle spindle contraction [62]. Besides this, eperisone has an analgesic effect, which apparently is mediated by inhibition of the release of substance P [63]. Accordingly, one may hypothesize that eperisone at least partially blocked the action of ESWs on substance P nerve fibers in this study, which may explain why rESWT was superior to rESWT + C + E in treatment of cnsLBP (when assessed using the NRS) in this study. This phenomenon should be addressed in future studies.

### Limitations

This study has a number of limitations. One limitation is the relatively high mean PSEQ score of the patients at baseline, which caused that the majority of patients could neither reach a point score change of 20 points which was defined as treatment success in the protocol of this study, nor a point score change of 11 points which has been defined in the literature as MCID of the PSEQ score when treating cnsLBP [32]. As outlined in detail above, this was due to the fact that the patients investigated in this study were not specifically selected but were representative for all patients who seek medical treatment of cnsLBP at our department (the latter is representative for departments of pain medicine at hospitals affiliated with university schools of medicine in China). On the other hand, a post-hoc subgroup analysis of those patients in this study with a PSEQ score < 50 (i.e., those patients who could have reached the MCID of the PSEQ score when treating cnsLBP) indicated that this study would have come to similar conclusions if patients would have been preselected (Additional file 3).

Another limitation is that only a single pharmacological treatment was tested in this study. Accordingly, the question could not be answered whether any combination of rESWT with a pharmacological treatment (NSAIDs and SMRs) or any pharmacological treatment alone could be superior to rESWT in treatment of cnsLBP. This should be addressed in future studies.

Finally, the protocol of this study did not allow to finally answer the question whether rESWT is non-inferior or even superior to rESWT + C + E or C + E alone. However, considering the potential negative interaction between ESWs and eperisome in treatment of cnsLBP this limitation appears irrelevant. Future studies on this topic will most probably not consider eperisome anymore in related combination therapies.

### Conclusions

This study suggests that the use of rESWT in patients with cnsLBP who seek medical treatment at a department of pain medicine in China is safe and effective, leading to a significant reduction in pain, without adverse events. In this regard this study corroborates similar findings from other parts of the world. Particularly in China clinicians should consider rESWT as a non-pharmacological alternative to celecoxib and eperisone in the management of cnsLBP. Based on apparently opposite effects on substance P nerve fibers the combination of rESWT and eperisone should not be considered anymore. Future studies should address whether the combination of rESWT with celexocib and/or other SMRs (including baclofen) is superior to rESWT or pharmacological treatment alone.

## Supplementary Information

**Additional file 1.** Studies on radial extracorporeal shock wave therapy and focused extracorporeal shock wave therapy for nonspecific low back pain that were published so far.

**Additional file 2.** Studies listed in the PEDro database in which the efficacy of non-pharmacological, conservative treatments in the management of low back pain was compared with the efficacy of pharmacological treatments and the combination thereof.

**Additional file 3.** Outcome of the present study of the subgroup of patients with Pain Self-Efficacy Questionnaire (PSEQ) score < 50 at baseline (these patients could have reached the minimal clinically important difference of the PSEQ score when treating chronic nonspecific low back pain).

## Data Availability

The datasets used and analyzed in this study are available from the corresponding author on reasonable request, taking into account any confidentiality.

## Abbreviations

A: Age
ANOVA: Analysis of variance
BH: Body height
BL: Baseline
BMI: Body mass index
BW: Body weight
C + E: Celecoxib and eperisone
CASP: Chinese Association for the Study of Pain
Ch: Change compared to baseline
cnsLBP: Chronic nonspecific low back pain
Co: Comorbidities
CONSORT: Consolidated Standards of Reporting Trials
Cox-2: Cyclooxygenase-2
Du: Duration
D0: Day zero
EFD: Energy flux density
ES: Education status
ESWs: Extracorporeal shock waves
ESWT: Extracorporeal shock wave therapy
fESWT: Focused extracorporeal shock wave therapy
G: Gender
L1/…/L5: First/…/fifth lumbar segment of the spine
LMU: Ludwig-Maximilians-University
max: Maximum value
MCID: Minimally clinically important difference
mg: Milligram
min: Minimum value
MRI: Magnetic resonance imaging
MS: Marriage status
n: Number
NRS: Numerical Rating Scale
NSAIDs: Nonsteroidal anti-inflammatory drugs
nsLBP: Nonspecific low back pain
OLBPDQ: Oswestry Low Back Pain Disability Questionnaire
PHQ-9: Patient Health Questionnaire 9
PSEQ: Pain Self-Efficacy Questionnaire
PubMed: Https://pubmed.ncbi.nlm.nih.gov/
RCTs: Randomized controlled trials
rESWs: Radial extracorporeal shock waves
rESWT: Radial extracorporeal shock wave therapy
rESWT + C + E: Combination of radial extracorporeal shock wave therapy, celecoxib and eperisone
S1/…/S5: First/…/fifth sacral segment of the spine
SD: Standard deviation
SMRs: Skeletal muscle relaxants
SPIRIT: Standard Protocol Items: Recommendations for Interventional Trials
T1/…/T12: First/…/twelfth thoracal segment of the spine
USA: United States of America
W: Week after baseline
W1/W2/W3/W4/W5/W10/W12: One week/two weeks/three weeks/four weeks/five weeks/ten weeks/twelve weeks after baseline
