# Supplementary material for: Efficacy and safety of treating chronic nonspecific low back pain with radial extracorporeal shock wave therapy (rESWT), rESWT combined with celecoxib and eperisone (C + E) or C + E alone: a prospective, randomized trial
Source: J Orthop Surg Res. 2021 Dec 4;16:705. doi: 10.1186/s13018-021-02848-x (PMC8642949; doi:10.1186/s13018-021-02848-x)
Supplement: Supplementary file 1 — Additional file 1. Studies on radial extracorporeal shock wave therapy and focused extracorporeal shock wave therapy for nonspecific low back pain that were published so far. [file 13018_2021_2848_MOESM1_ESM.docx]

**Efficacy and safety of treating chronic nonspecific low back pain with radial extracorporeal shock wave therapy (rESWT), rESWT combined with celecoxib and eperisone (C+E) or C+E alone: a prospective, randomized trial**

X. Guo, L. Li, Z. Yan, Y. Li, Z. Peng, Y. Yang, Y. Zhang, C. Schmitz, Z. Feng

**Additional File 1** Studies on radial extracorporeal shock wave therapy (rESWT) and focused extracorporeal shock wave therapy (fESWT) for nonspecific low back pain (LBP) that were published so far. Abbreviations in Line *Outcome*: >, the former therapy was more effective than the latter therapy; ≈, the effectiveness of both therapies was approximately the same.

| **Study** | **Nedelka et al. [1]** | **Lee et al. [2]** | **Han et al. [3]** |
| --- | --- | --- | --- |
| Diagnosis | Unilateral chronic lumbar facet pain | Chronic LBP | Chronic LBP |
| Type of study | Pilot retrospective study | Cohort study (no randomization) | Cohort study (no randomization) |
| Power analysis | Not reported | Not reported | Not reported |
| No. of subjects | 21 | 13 | 15 |
| Device | Duolith, radial part (Storz Medical, Tägerwillen, Switzerland) | JEST-2000 (Joeun Medical, Daejeon, Korea | VITERA (Comed, Korea) |
| Type of ESWT | rESWT | rESWT | fESWT, electrohydraulic |
| Applicator | 15-mm titanium DPI applicator | 17-mm head | 17-mm head |
| No. of ESWT sessions | 5 | 12 | 12 |
| Interval between ESWT sessions [days] | 7 | 3.5 | 3.5 |
| No. of shock waves per ESWT session | 3000 | 2000 | 1000 |
| Bar / EFD of shock waves | 3.5 /0.12 mJ/mm2 | 0.10 mJ/mm2 | 0.01-0.16 mJ/mm2 |
| Frequency of shock waves [Hz] | Not provided | 7 | 7 |
| Control treatment 1 |  |  |  |
| No. of subjects | 20 | 15 | 15 |
| Procedure | Single injection of 6 ml 1% trimecaine and 7 mg of betamethazone (C1) | Hyperthermia using hot packs, ultrasound and TENS (C) | Hyperthermia using hot packs (20 min), ultrasound (5 min) and TENS (15 min) (C) |
| Control treatment 2 |  |  |  |
| No. of subjects | 20 |  |  |
| Procedure | Radiofrequency Medial Branch Neurotomy (C2) |  |  |
| Follow-up interval | M2, M6 and M12 | Not provided | Not provided |
| Investigated variables | Pain (VAS score), Oswestry Disability Index (ODI) | Pain (VAS score), dynamic balance ability | Pain (VAS score), ODI, Beck depression index |
| Definition of treatment success | Not provided | Not provided | Not provided |
| VAS at baseline (ESWT) | Not provided | 7.2 ± 0.8 (SD or SEM) | 7.0 ± 0.76 (SD or SEM) |
| VAS at follow-up (ESWT) | Not provided | 4.5 ± 1.1 (SD or SEM) | 3.6 ± 1.1 (SD or SEM) |
| Outcome | C2 > rESWT > C1 | rESWT > C | fESWT > C |

**Additional File 1 (cont.)**

| **Study** | **Moon et al. [4]** | **Notarnicola et al. [5]** | **Tomska et al. [6]** |
| --- | --- | --- | --- |
| Diagnosis | Sacroiliac joint pain | LBP | Back pain |
| Type of study | RCT | RCT | RCT |
| Power analysis | Not reported | Not reported | Not reported |
| No. of subjects | 15 | ? (total 30) | 36 |
| Device | Aries (Dornier MedTech, Wessling, Germany) | Minilith SL1 (Storz Medical) | Not reported |
| Type of ESWT | fESWT | fESWT | rESWT |
| Applicator | --- | --- | Not reported |
| No. of ESWT sessions | 1 | 3 | 6 |
| Interval between ESWT sessions [days] | N/a | 7 | 7 |
| No. of shock waves per ESWT session | 2000 | 2000 | 2000 |
| Bar / EFD of shock waves | 0.09-0.25 mJ/mm2 (maximum tolerated by the patient) | 0.03 mJ/mm2 | 60-100 mJ |
| Frequency of shock waves [Hz] | 3 | Not reported | 5-10 |
| Control treatment (C) |  |  |  |
| No. of subjects | 15 | ? (total 30) | 37 |
| Procedure | Sham ESWT | Individual exercise treatment | Deep electromagnetic stimulation |
| Follow-up interval | W1 and W4 | M1 and M3 | W0, W2 and W4 after treatment |
| Investigated variables | Pain (VAS score), ODI | Pain (VAS score), Roland Morris Disability Questionnaire, ODI | Pain (VAS score) |
| Definition of treatment success | Not provided | Not provided | Not provided |
| VAS at baseline (ESWT) | 6.42 ± ? | Not provided | 6.8 ± 2.3 (Mean ± SD) |
| VAS at follow-up (ESWT) | 3.64 ± ? | Not provided | 2.6 ± 2.0 (Mean ± SD) |
| Outcome | fESWT > C | ESWT > C | rESWT ≈ C |

**Additional File 1 (cont.)**

| **Study** | **Walewicz et al. [7]** | **Walewicz et al. [8]** | **Eftekharsadat et al. [9]** |
| --- | --- | --- | --- |
| Diagnosis | Chronic LBP | Chronic LBP (discopathy of the L5-S1 spine segment) | Inferior trigger points in the quadratus lumborum muscle |
| Type of study | RCT | RCT | RCT |
| Power analysis | Yes | Not reported | Yes |
| No. of subjects | 20 | 20 | 27 |
| Device | Pro-Shock Waves (Cosmogamma, Indonesia) | Cellactor (Storz Medical) | enPulsPro (Zimmer MedizinSysteme, Neu-Ulm, Germany) |
| Type of ESWT | rESWT | rESWT | rESWT |
| Applicator | Not reported | Not reported | Not reported |
| No. of ESWT sessions | 10 | 10 | 5 |
| Interval between ESWT sessions [days] | 3.5 | 3.5 | 7 |
| No. of shock waves per ESWT session | 2000 | 2000 | 1500 |
| Bar / EFD of shock waves | 2.5 / 0.1 mJ/mm2 | 2.5 / --- | 0.1 mJ/mm2 |
| Frequency of shock waves [Hz] | 5 | 5 | 10-16 |
| Additional treatment | Functional training | Core stability treatment | Stretching exercises, education, acetaminophen |
| Control treatment (C) |  |  |  |
| No. of subjects | 20 | 20 | 27 |
| Procedure | Sham ESWT | Sham ESWT | Injection of corticosteroid |
| Follow-up interval | M1 and M3 | M1 and M3 | W2, W4 |
| Investigated variables | Pain (VAS score), Laitinen Pain Scale, ODI | Pain (Laitinen Pain Scale), Roland–Morris Questionnaire, original Schober Test, assessment of postural sway | Pain (VAS score), Pressure-pain threshold, ODI, Quality of Life |
| Definition of treatment success | Not provided | Not provided | Not provided |
| VAS at baseline (ESWT) | 4.7 ± 1.9 (Mean ± SD) | Not provided | 7.63 ± 0.27 (Mean ± SD) |
| VAS at follow-up (ESWT) | 2.0 ± 2.0 (Mean ± SD) | Not provided | 5.11 ± 0.36 (Mean ± SD) |
| Outcome | rESWT > C | rESWT > C | rESWT ≈ C |

**Additional File 1 (cont.)**

| **Study** | **Kong et al. [10]** | **Celik et al. [11]** |
| --- | --- | --- |
| Diagnosis | Chronic LBP | Chronic LBP |
| Type of study | RCT | RCT |
| Power analysis | No | No |
| No. of subjects | 100 | 25 |
| Device | HK. ESWO-AJ (Shenzhen Huikang Medical Apparatus Co., Ltd., Shenzen, Chaina) | E1000 (EMD Medical Technologies, Ankara, Turkey) |
| Type of ESWT | fESWT | fESWT |
| Applicator | --- | --- |
| No. of ESWT sessions | 5-10 | 12 |
| Interval between ESWT sessions [days] | Not provided | 3.5 |
| No. of shock waves per ESWT session | 1200 | 1500 |
| Bar / EFD of shock waves | 0.1-0.2 mJ/mm^2^ | 0.12 mJ/mm^2^ |
| Frequency of shock waves [Hz] | Not provided | 2.5 |
| Control treatment (C) |  |  |
| No. of subjects | 100 | 25 |
| Procedure | Laser therapy | Sham ESWT |
| Follow-up interval | Not provided | W6, W12 |
| Investigated variables | Pain (VAS score), Quality of life | Pain (VAS score), ODI, Hospital Anxiety and Depression Scale, Short-form 36 |
| Definition of treatment success | Not provided | Not provided |
| VAS at baseline (ESWT) | 6.3 ± 1.1 (SD or SEM) | 5 (2-8) (median (range)) |
| VAS at follow-up (ESWT) | Not provided | 0 (0-7) (median (range)) |
| Outcome | fESWT > C | fESWT > C |

**References:**

1. Nedelka T, Nedelka J, Schlenker J, Hankins C, Mazanec R. Mechano-transduction effect of shockwaves in the treatment of lumbar facet joint pain: comparative effectiveness evaluation of shockwave therapy, steroid injections and radiofrequency medial branch neurotomy. Neuro Endocrinol Lett. 2014;35(5):393-7.

2. Lee S, Lee D, Park J. Effects of extracorporeal shockwave therapy on patients with chronic low back pain and their dynamic balance ability. J Phys Ther Sci. 2014;26(1):7-10. doi: 10.1589/jpts.26.7.

3. Han H, Lee D, Lee S, Jeon C, Kim T. The effects of extracorporeal shock wave therapy on pain, disability, and depression of chronic low back pain patients. J Phys Ther Sci. 2015;27(2):397-9. doi: 10.1589/jpts.27.397.

4. Moon YE, Seok H, Kim SH, Lee SY, Yeo JH. Extracorporeal shock wave therapy for sacroiliac joint pain: A prospective, randomized, sham-controlled short-term trial. J Back Musculoskelet Rehabil. 2017;30(4):779-84. doi: 10.3233/BMR-150405.

5. Notarnicola A, Maccagnano G, Gallone MF, Mastromauro L, Rifino F, Pesce V, Covelli I, Moretti B. Extracorporeal shockwave therapy versus exercise program in patients with low back pain: short-term results of a randomised controlled trial. J Biol Regul Homeost Agents. 2018;32(2):385-9.

6. Tomska N, Turoń-Skrzypińska A, Szylińska A, Rył A, Lubińska-Gruszka A, Mosiejczuk H, Rotter I. Deep electromagnetic stimulation and radial shock wave therapy in back pain. Ortop Traumatol Rehabil. 2018;20(3):189-95. doi: 10.5604/01.3001.0012.0943.

7. Walewicz K, Taradaj J, Rajfur K, Ptaszkowski K, Kuszewski MT, Sopel M, Dymarek R. The effectiveness of radial extracorporeal shock wave therapy in patients with chronic low back pain: a prospective, randomized, single-blinded pilot study. Clin Interv Aging. 2019;14:1859-69. doi: 10.2147/CIA.S224001.

8. Walewicz K, Taradaj J, Dobrzyński M, Sopel M, Kowal M, Ptaszkowski K, Dymarek R. Effect of radial extracorporeal shock wave therapy on pain intensity, functional efficiency, and postural control parameters in patients with chronic low back pain: a randomized clinical trial. J Clin Med. 2020;9(2):568. doi: 10.3390/jcm9020568.

9. Eftekharsadat B, Fasaie N, Golalizadeh D, Babaei-Ghazani A, Jahanjou F, Eslampoor Y, Dolatkhah N. Comparison of efficacy of corticosteroid injection versus extracorporeal shock wave therapy on inferior trigger points in the quadratus lumborum muscle: a randomized clinical trial. BMC Musculoskelet Disord. 2020;21(1):695. doi: 10.1186/s12891-020-03714-3.

10. Kong L, Tian X, Yao X. Effects of extracorporeal shock wave therapy on chronic low back pain and quality of life. Revista Argent Clin Psicol. 2020,20,883-7. doi: 10.24205/03276716.2020.896.

11. Çelik A, Altan L, Ökmen BM. The effects of extracorporeal shock wave therapy on pain, disability and life quality of chronic low back pain patients. Altern Ther Health Med. 2020;26(2):54-60.
