# Supplementary material for: Efficacy and safety of treating chronic nonspecific low back pain with radial extracorporeal shock wave therapy (rESWT), rESWT combined with celecoxib and eperisone (C + E) or C + E alone: a prospective, randomized trial
Source: J Orthop Surg Res. 2021 Dec 4;16:705. doi: 10.1186/s13018-021-02848-x (PMC8642949; doi:10.1186/s13018-021-02848-x)
Supplement: Supplementary file 2 — Additional file 2. Studies listed in the PEDro database in which the efficacy of non-pharmacological, conservative treatments in the management of low back pain was compared with the efficacy of pharmacological treatments and the combination thereof. [file 13018_2021_2848_MOESM2_ESM.docx]

**Efficacy and safety of treating chronic nonspecific low back pain with radial extracorporeal shock wave therapy (rESWT), rESWT combined with celecoxib and eperisone (C+E) or C+E alone: a prospective, randomized trial**

X. Guo, L. Li, Z. Yan, Y. Li, Z. Peng, Y. Yang, Y. Zhang, C. Schmitz, Z. Feng

**Additional File 2** Studies listed in the PEDro database [1] in which the efficacy of non-pharmacological, conservative treatments in the management of low back pain (LBP) was compared with the efficacy of pharmacological treatments and the combination thereof. Abbreviations, W, week; SD, standard deviation; >, the former therapy was more effective than the latter therapy.

*,respectively diazepam (5 mg), Hartmann's solution (5% dextrose in 0.9% NaCl), paracetamol (1 g), paracetamol (500 mg) + codeine (30 mg), tramadol (50-100 mg), dextropropoxyphene (32.5 mg) + paracetamol (325 mg) or ibuprofen (400 mg) + diclofenac (50 mg) + indomethacin (100 mg) as needed; single application.

| **Study** | **Hancock et al. [2]** | **Cohen et al. [3]** | **Zaringhalam et al. [4]** |
| --- | --- | --- | --- |
| Diagnosis | Acute LBP | Acute LBP, migraine or ankle sprain | Chronic nonspecific LBP |
| Type of study | RCT | RCT | RCT |
| No. of subjects | 239 | 528 | 84 |
| Follow-up | W12 | 1 hour | W5, W10 |
| Treatment 1 (non-pharmacological, conservative) | Spinal manipulation (2-3 treatment sessions per week for up to 4 weeks) + placebo drug treatment | Acupuncture | Spinal manipulation (2-3 treatment sessions per week for up to 4 weeks) + placebo drug treatment |
| N | 59 | 177 | 21 |
| VAS at baseline | Not provided | 8.5 ± 1.6 (mean ± SD) | 6.4 ± 1.8 (mean ± SD) |
| VAS at follow-up | Not provided | 6.6 ± 2.6 (mean ± SD) | W5: 4.47 ± 1.9  W10: 5.0 ± 2.0 |
| Treatment 2 (combination) | Spinal manipulation + Paracetamol | Acupuncture + pharmacotherapy | Acupuncture + baclofen |
| N | 60 | 178 | 21 |
| VAS at baseline | Not provided | 8.5 ± 1.6 (mean ± SD) | 6.5 ± 1.7 (mean ± SD) |
| VAS at follow-up | Not provided | 6.3 ± 2.8 (mean ± SD) | W5: 4.0 ± 1.3  W10: 4.7 ± 1.4 |
| Treatment 3 (pharmacological) | Placebo spinal manipulation + Paracetamol (4 × 1g/day for up to 4 weeks) | Pharmacotherapy* | Baclofen  (2 × 15 mg/day for 5 weeks) |
| N | 60 | 173 | 21 |
| VAS at baseline | Not provided | 8.5 ± 1.6 (mean ± SD) | 6.5 ± 1.8 (mean ± SD) |
| VAS at follow-up | Not provided | 6.5 ± 2.7 (mean ± SD) | W5: 6.2 ± 2.2  W10: 6.4 ± 2.4 |
| Treatment 4 (additional) | Placebo spinal manipulation + placebo drug treatment | --- | No pain reduction treatment |
| N | 60 |  | 21 |
| VAS at baseline | Not provided |  | 6.5 ± 1.9 (mean ± SD) |
| VAS at follow-up | Not provided |  | W5: 6.4 ± 2.4  W10: 6.4 ± 2.6 |
| Outcome | --- | --- | 1 > 3 and 1 > 4  2 > 3 and 2 > 4 |

**References:**

1. www.pedro.org.au [cited 07 December 2020].

2. Hancock MJ, Maher CG, Latimer J, McLachlan AJ, Cooper CW, Day RO, Spindler MF, McAuley JH. Assessment of diclofenac or spinal manipulative therapy, or both, in addition to recommended first-line treatment for acute low back pain: a randomised controlled trial. Lancet. 2007;370(9599):1638-43. doi: 10.1016/S0140-6736(07)61686-9.

3. Cohen MM, Smit V, Andrianopoulos N, Ben-Meir M, Taylor DM, Parker SJ, Xue CC, Cameron PA. Acupuncture for analgesia in the emergency department: a multicentre, randomised, equivalence and non-inferiority trial. Med J Aust. 2017;206(11):494-9. doi: 10.5694/mja16.00771.

4. Zaringhalam J, Manaheji H, Rastqar A, Zaringhalam M. Reduction of chronic non-specific low back pain: a randomised controlled clinical trial on acupuncture and baclofen. Chin Med. 2010;5:15. doi: 10.1186/1749-8546-5-15.
