# Supplementary material for: Efficacy and safety of treating chronic nonspecific low back pain with radial extracorporeal shock wave therapy (rESWT), rESWT combined with celecoxib and eperisone (C + E) or C + E alone: a prospective, randomized trial
Source: J Orthop Surg Res. 2021 Dec 4;16:705. doi: 10.1186/s13018-021-02848-x (PMC8642949; doi:10.1186/s13018-021-02848-x)
Supplement: Supplementary file 3 — Additional file 3. Outcome of the present study of the subgroup of patients with Pain Self-Efficacy Questionnaire (PSEQ) score < 50 at baseline (these patients could have reached the minimal clinically important difference of the PSEQ score when treating chronic nonspecific low back pain). [file 13018_2021_2848_MOESM3_ESM.docx]

**Efficacy and safety of treating chronic nonspecific low back pain with radial extracorporeal shock wave therapy (rESWT), rESWT combined with celecoxib and eperisone (C+E) or C+E alone: a prospective, randomized trial**

X. Guo, L. Li, Z. Yan, Y. Li, Z. Peng, Y. Yang, Y. Zhang, C. Schmitz, Z. Feng

**Additional File 3** Outcome of the present study of the subgroup of subjects with Pain Self-Efficacy Questionnaire (PSEQ) score < 50 at baseline (radial extracorporeal shock wave therapy (rESWT): n=16; rESWT combined with celecoxib and eperisone (C+E) (n=21); C+E alone: n=20) (these subjects could have reached the minimal clinically important difference of the PSEQ score when treating chronic nonspecific low back pain). The figure shows Tukey boxplots of (**A**) PSEQ score, (**B**) Numerical Rating Scale (NRS) score, (**C**) Oswestry Low Back Pain Disability Questionnaire (OLBPDQ) score and (**D**) Patient Health Questionnaire 9 (PHQ-9) score of subjects suffering from chronic low back pain who were treated with respectively rESWT (dark gray bars), rESWT+C+E (light gray bars) or C+E alone (open bars) at baseline (X=0) and different follow-up times. The table summarizes key results of the statistical analysis of these data using two-way repeated measures ANOVA (P values < 0.05 are given boldface). Bonferroni's multiple comparison test showed no statistically significant differences between groups.


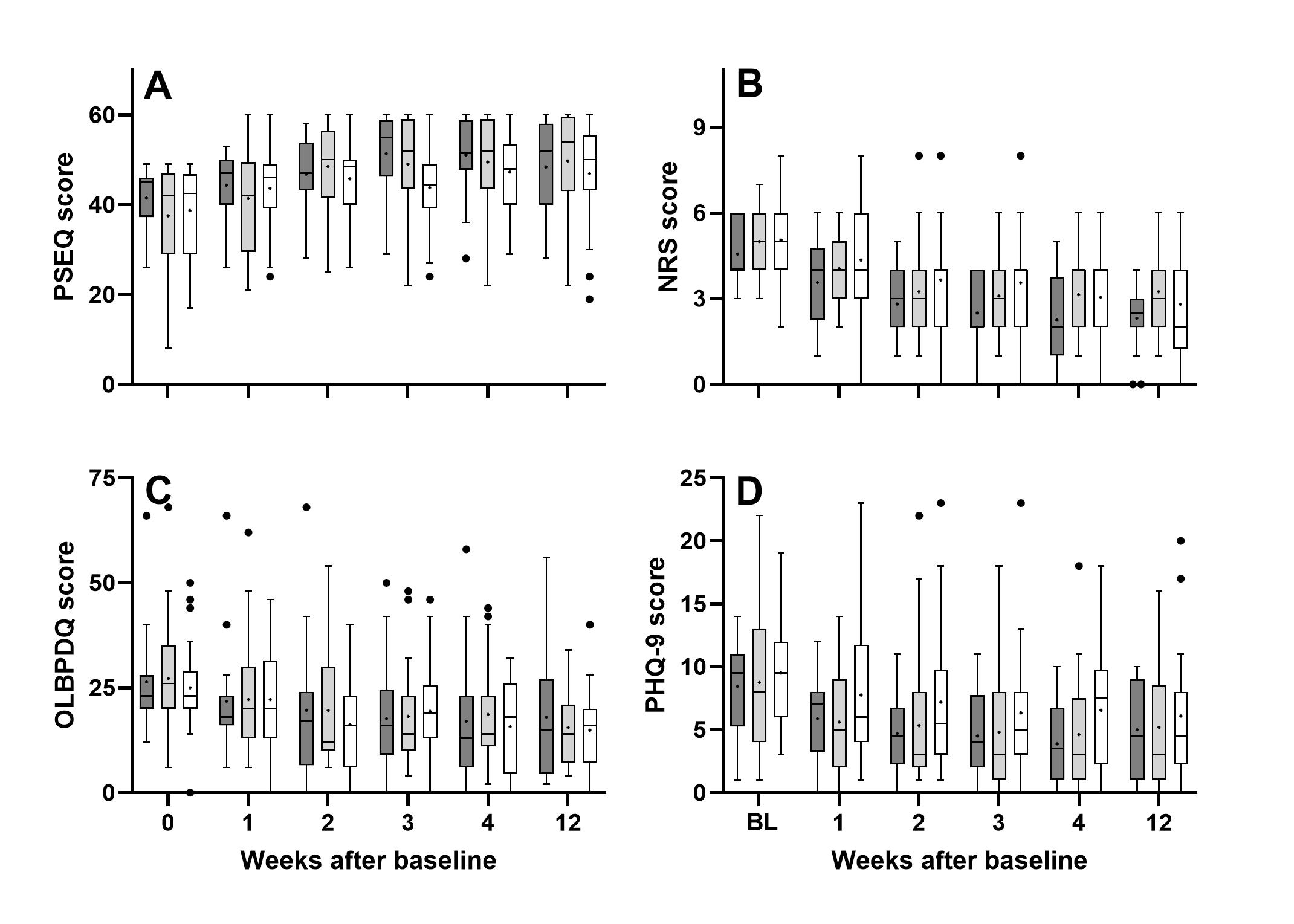


| **ANOVA: source of variation** | **PSEQ score** | **NRS score** | **OLBPDQ score** | **PHQ-9 score** |
| --- | --- | --- | --- | --- |
| Time | **<0.001** | **<0.001** | **<0.001** | **<0.001** |
| Treatment | 0.550 | 0.200 | 0.926 | 0.339 |
| Time × Treatment | 0.195 | 0.666 | 0.677 | 0.933 |
| Subject | **<0.001** | **<0.001** | **<0.001** | **<0.001** |
